# Oxidative Stress and Male Infertility: A Critical Analysis of Current Diagnostic and Therapeutic Methods

**DOI:** 10.3390/antiox15080937

**Published:** 2026-07-28

**Authors:** Jorge Hallak, Robert John Aitken, João Sallum Hallak, Ana Flávia Quiarato Lozano, Thiago Afonso Teixeira

**Affiliations:** 1 Division of Urology Department of Surgery, University of São Paulo Medical School, São Paulo 01246-000, Brazil; 2 Department of Pathology, Reproductive Toxicology Unit, University of São Paulo Medical School, São Paulo 01246-000, Brazil; 3 Men’s Health Study Group, Institute for Advanced Studies, University of São Paulo, São Paulo 01230-000, Brazil; thafonsoteixeira@gmail.com; 4 Androscience, Science and Innovation Center in Andrology Laboratories, High-Complex Clinical and Research Laboratory, and the Androscience Institute for Science, Education and Advanced Projects in Male Health, São Paulo 04534-011, Brazil; joao.hallak@aluno.fmabc.net (J.S.H.); anaquiarato@gmail.com (A.F.Q.L.); 5 Priority Research Centre for Reproductive Science, University of Newcastle, Callaghan, NSW 2308, Australia; john.aitken@newcastle.edu.au; 6 FMABC Medical School University Center, Santo André 09060-060, Brazil; 7 Division of Urology, Hospital Universitário, School of Medicine, Federal University of Amapá, Macapá 68903-419, Brazil

**Keywords:** male infertility, oxidative stress, reactive oxygen species, antioxidants, sperm DNA fragmentation, lipid peroxidation, semen analysis, male reproductive health, redox biology

## Abstract

Male infertility affects a substantial proportion of couples worldwide, with oxidative stress recognized as one of its most prevalent and potentially treatable mechanisms. This review critically analyzes current diagnostic and therapeutic approaches for oxidative stress-related male infertility, highlighting the necessity of advancing from descriptive semen analysis to mechanism-based clinical care. The biological basis of reactive oxygen species generation in spermatozoa, the dual physiological and pathological roles of redox signaling, and the contributions of leukocytes, lifestyle factors, infections, varicocele, metabolic disease, environmental exposures, and transition metals in amplifying oxidative injury are examined. Laboratory methods for assessing oxidative stress, including chemiluminescence, flow cytometry, oxidation-reduction potential, and total antioxidant capacity assays, are evaluated with respect to biological relevance, technical limitations, standardization, and clinical applicability. Evidence suggests that indiscriminate antioxidant use, without prior confirmation of oxidative stress or identification of its cause, may account for inconsistent clinical trial outcomes and could induce reductive stress. Conversely, individualized management based on comprehensive andrological evaluation, treatment of underlying conditions, and quality-controlled functional sperm testing is more rational and potentially effective. Oxidative stress should therefore be regarded as a diagnosable and monitorable clinical entity in male infertility, necessitating validated diagnostics and personalized therapeutic strategies rather than empiric supplementation alone.

## 1. Introduction

Male infertility affects circa 10% of all men and contributes to approximately 45% to 52% of infertility cases, with global sperm concentrations and total sperm count declining by 51.6% and 62.3%, respectively, over the last five decades. This progressive deterioration of male reproductive health has been observed in all four Continents and has driven the scientific community to urgently address the causes and cofactors involved, in the hope of finding new avenues for reversing this negative trend [1,2,3]. Although the World Health Organization has standardized and, in recent editions, simplified semen analysis protocols to ensure universal uniformity and provide a global framework for classification, it remains fundamentally descriptive and fails to address sperm function and etiological complexity [2,4]. Consequently, clinical management frequently defaults to assisted reproductive technologies, particularly intracytoplasmic sperm injection (ICSI), bypassing diagnosis of the underlying causes and focusing exclusively on the applications of advanced technological tools [5]. In this context, oxidative stress (OS) has emerged as a central, unifying, and potentially reversible mechanism linking intrinsic (e.g., varicocele, metabolic, inflammatory, and hormonal imbalances, drugs and medications, infections) and extrinsic (e.g., environmental) factors affecting male fertility [6]. Unlike genetic and epigenetic defects, which are largely irreversible or progressive, OS represents a modifiable target, positioning redox biology at the core of translational andrology [7].

## 2. Redox Biology of Spermatozoa: Functional Necessity Versus Vulnerability

Reactive oxygen species (ROS) are indispensable regulators of sperm function, mediating capacitation, hyperactivation, and acrosomal exocytosis through tightly controlled redox signaling pathways [6]. ROS stimulate adenylyl cyclase, inhibit tyrosine phosphatases, and promote phosphorylation cascades essential for fertilization competence. Concurrently, ROS facilitates cholesterol efflux and membrane remodeling, enabling capacitation-associated increases in membrane fluidity [8,9,10]. Lipid-derived signaling cascades further integrate ROS into reproductive function, with prostaglandin synthesis driving CatSper-dependent calcium influx and acrosomal activation [11,12,13,14]. During epididymal maturation, ROS-mediated disulfide bond formation stabilizes protamine-packed chromatin, enhancing DNA integrity and mechanical resilience [8,9].

**Figure 1:**
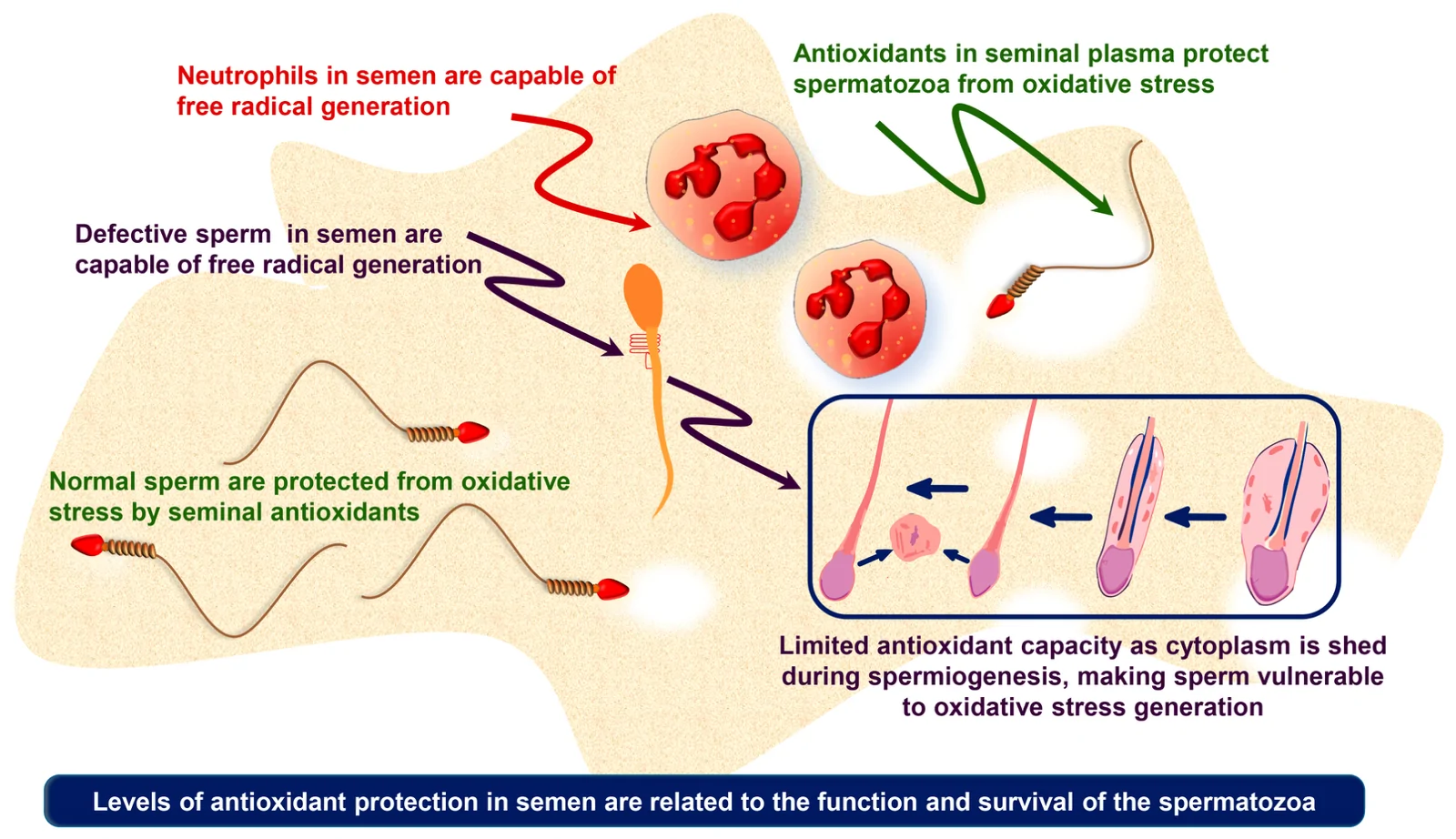
The impact of oxidative stress and antioxidant protection on spermatozoa. Neutrophils in semen serve as a primary source of reactive oxygen species (ROS), as shown by red arrows. Defective spermatozoa (purple arrow) also produce ROS, thereby increasing oxidative stress. In contrast, normal spermatozoa (green arrow) are protected by antioxidants present in seminal plasma. The inset demonstrates spermiogenesis, during which progressive cytoplasmic extrusion diminishes intrinsic antioxidant reserves, making mature spermatozoa more susceptible to oxidative injury. Blue arrows illustrate the sequential stages of maturation and the physiological release or pathological retention of the cytoplasmic droplet, with the latter associated with increased vulnerability to ROS-mediated damage.

This physiological reliance on ROS is counterbalanced by extreme structural vulnerability. Sperm membranes are enriched in polyunsaturated fatty acids, particularly docosahexaenoic acid, rendering them highly susceptible to lipid peroxidation [15,16]. Moreover, cytoplasmic extrusion during spermiogenesis markedly reduces intracellular antioxidant capacity, making spermatozoa dependent on extracellular antioxidant systems within seminal plasma [15], which is why human seminal plasma is such a rich source of antioxidants, as shown in Figure 1 and Table 1. Thus, spermatozoa operate within a narrow redox window, where slight perturbations shift ROS from essential signaling molecules to mediators of cellular damage.

Oxidative stress represents a major etiological factor in male infertility, with clinical data demonstrating its involvement in 35% to 87% of male factor subfertility cases [17]. Given that male factors account for a little more than 50% of all infertility cases in couples, and that oxidative stress has been identified as a pathological mechanism in approximately half of infertile males, the clinical burden of OS-induced reproductive dysfunction is substantial [18,19]. The vulnerability of spermatozoa to oxidative damage stems from their unique cellular architecture: during the final stages of spermatogenesis, most of the cytoplasm—and consequently, antioxidant defense systems—is discarded, rendering mature spermatozoa particularly susceptible to ROS-mediated injury [18]. This structural limitation, combined with the high polyunsaturated fatty acid content of sperm membranes, creates a cellular environment where even modest increases in ROS can trigger cascading oxidative damage, ultimately impairing sperm motility, inducing DNA fragmentation, and compromising fertilization capacity [20]. These epidemiological and mechanistic data underscore the critical importance of developing precise diagnostic strategies and mechanism-based therapeutic interventions to address oxidative stress in male infertility.

## 3. Source and Amplification of Oxidative Stress

### 3.1. Intrinsic ROS Generation

Endogenous ROS production arises primarily from mitochondrial electron leakage and NADPH oxidase (NOX) activity, particularly NOX5 [21]. Under physiological conditions, mitochondrial ROS generation supports sperm function; however, pathological stimuli—including thermal stress, electromagnetic radiation, and excess unsaturated fatty acids—enhance electron leakage and superoxide formation [22]. Additional contributors to mitochondrial ROS generation include certain polyphenolic food supplements [23], environmental toxicants such as phthalate esters [24], and soluble bacterial products, such as those from Escherichia coli [25]. Factors that activate the intrinsic apoptotic cascade [26], such as prolonged storage in vivo due to low ejaculation frequency [27], or in vitro during cryopreservation [28], also promote mitochondrial ROS production.

Defective spermiogenesis further exacerbates ROS production by retaining residual cytoplasm, increasing NADPH availability, and fueling oxidase-driven ROS generation [29,30]. Quantitative data demonstrate positive associations between ROS generation and cytoplasmic enzymes, such as SOD, creatine kinase, and G-6-PDH, all of which are inversely associated with sperm motility [31]. NOX5 overexpression has also been consistently associated with impaired motility and abnormal morphology, linking intrinsic redox dysregulation to clinical infertility phenotypes, particularly asthenozoospermia [29,30].

### 3.2. Extrinsic ROS Generation

Extracellular ROS are predominantly generated by leukocytes present in semen, particularly activated neutrophils and macrophages. While physiologically present, leukocyte activation dramatically amplifies the oxidative burden during infection, inflammation, and systemic disease. Thus, multiple pro-inflammatory clinical conditions—including varicocele, obesity, diabetes, smoking, psychological stress, aging, and infection, including those caused by the Severe Acute Respiratory Syndrome Coronavirus 2 (SARS-CoV-2)—converge on oxidative pathways, reinforcing OS as a common downstream mechanism in male infertility [32,33,34,35,36,37,38,39,40,41].

Accumulating evidence indicates that oxidative stress is a major mediator of the adverse effects of SARS-CoV-2 infection on male reproductive function. In a seminal ultrastructural study, Hallak et al. hitherto demonstrated the persistence of intracellular SARS-CoV-2 particles within ejaculated spermatozoa for up to 110 days after infection and described a previously unrecognized ETosis-like response termed sperm extracellular trap-like (SETs-L) [41]. The authors proposed that the intense inflammatory milieu generated by COVID-19 drives excessive ROS production in the male reproductive tract, resulting in oxidative damage, chromatin destabilization, membrane disruption, and the extrusion of extracellular sperm DNA that can entrap viral particles, emphasizing the role of the spermatozoa not only as a reproductive-specialized cell but also as a component of the immune system activated under critical circumstances. These observations are consistent with the well-established role of ROS in triggering extracellular trap formation in immune cells and suggest that spermatozoa may activate an evolutionary maintained stress-like response under conditions of severe redox imbalance and external aggression, like the one provoked by the SARS-CoV-2 infection. Importantly, such mechanisms provide a biologically plausible explanation for impaired sperm motility, defective sperm function, and increased susceptibility to DNA damage reported following SARS-CoV-2 infection, reinforcing the concept that oxidative stress is a central pathway linking COVID-19-associated inflammation to male infertility, potentially opening new avenues for diagnosis and treatment strategies for acute or chronic viral infectious diseases or related to the ongoing long-COVID [40,41].

### 3.3. Self-Propagating Oxidative Damage

It is important to note that both intrinsic and extrinsic factors capable of generating oxidative stress in human spermatozoa only need to initiate the process for it to persist. Once lipid peroxidation begins, it proceeds as a chain reaction. Peroxyl and alkoxyl radicals propagate this chain by abstracting hydrogen atoms from adjacent lipids, generating carbon-centered radicals that react with oxygen to form lipid peroxides. This perpetuates the peroxidation cascade. Furthermore, electrophilic aldehydes produced during peroxidation form adducts with proteins in the mitochondrial electron transport chain, resulting in electron leakage and furthering ROS production in a self-amplifying oxidative cycle [42]. This self-perpetuating mechanism explains the persistence of oxidative damage even after removal of the initial insult, underscoring the importance of early intervention.

## 4. Transition Metals as Redox Amplifiers

Redox-active transition metals, particularly iron and copper, critically amplify OS through Fenton chemistry, converting hydrogen peroxide into highly reactive hydroxyl radicals, as depicted in the formula below [43,44,45]:

Fenton reaction with iron:

Fe(II) + H_2_O_2_ → Fe(III) + ^•^OH + OH^−^

Under physiological conditions, metal homeostasis is tightly regulated via transporters, storage proteins, and antioxidant systems [43,44,46,47,48]. However, disruption of metal regulation—through inflammation, metabolic imbalance, or cellular damage—enhances ROS generation and accelerates lipid peroxidation [43,44,45]. In spermatozoa, these processes exacerbate oxidative injury, acting as amplifiers rather than primary initiators of redox imbalance [45].

## 5. Clinical Assessment: The Translational Gap

### 5.1. Failure of Empirical Antioxidant Therapy

Despite strong mechanistic evidence, antioxidant therapies have yielded inconsistent clinical outcomes, largely because of inadequate patient selection. Instead of focusing on the OS status [49], participants are chosen based on clinic attendance, low sperm motility, or high DNA fragmentation, with few receiving a thorough andrological assessment—including clinical history, comorbidities, cofactors, detailed anamnesis, and examination of the external genitalia—to determine accurate diagnoses or trial eligibility. There is often little evidence that these patients have ROS-mediated pathology. If ROS-mediated cellular damage is not present, antioxidants are unlikely to be effective. Administering strong antioxidants in such cases risks causing reductive stress, which is as harmful as OS [50]. This raises a key issue: why do such ineffective practices persist?

### 5.2. Diagnostic Approaches

#### 5.2.1. Relationship Between Oxidative Stress Biomarkers and Male Infertility Outcome

Oxidative stress biomarkers have demonstrated significant associations with multiple dimensions of male reproductive dysfunction, establishing their clinical relevance beyond conventional basic semen parameters. Sperm DNA fragmentation (SDF), a downstream consequence of oxidative damage, shows strong correlations with reduced clinical pregnancy rates, live birth rates, and augmented miscarriage rates following intrauterine insemination (IUI), in vitro fertilization (IVF), and intracytoplasmic sperm injection (ICSI). Importantly, men with elevated SDF may present with normal conventional semen parameters yet experience poor natural conception rates, highlighting the diagnostic gap that oxidative biomarkers can address [51].

Seminal plasma free thiol levels, representing extracellular redox status, correlate positively with sperm concentration (r = 0.383, *p* = 0.008) and progressive motility (r = 0.333, *p* = 0.022). Similarly, malondialdehyde (MDA), a lipid peroxidation product, shows a positive correlation with sperm concentration (r = 0.314, *p* = 0.031). The biomarker 8-hydroxy-2′-deoxyguanosine (8-OHdG), a marker of DNA oxidation, is associated with semen quality, leukocytospermia, smoking status, and body mass index [52].

Recent evidence supports the concept of redox endophenotyping to reclassify idiopathic male infertility—which accounts for 30–40% of male factor cases—into mechanistically distinct categories including hyper-oxidative, hypo-oxidative, DNA damage-dominant, mitochondrial dysfunction, epigenetic-redox, and inflammatory-redox phenotypes [53,54]. Each endophenotype integrates specific biomarkers with distinct functional impairments and clinical outcomes, enabling personalized diagnostic and therapeutic strategies [53]. In the clinical andrology setting, the precise primary, secondary, and even tertiary reasons for each individual redox imbalance are imperative to determine whether it represents a treatable, untreatable condition, or both, with the most prevalent reason being the primary one. By adopting this rationale, the initial treatment strategy, and most importantly, the follow-up therapeutic use of antioxidants is more assertive with potentially more consistent results.

#### 5.2.2. Comprehensive Diagnostic Framework for Oxidative Stress-Induced Male Infertility

A comprehensive diagnostic approach to OS-induced male infertility extends beyond isolated ROS measurement to encompass clinical evaluation, conventional semen analysis, targeted OS biomarker assessment, and functional sperm testing.

The diagnostic process begins with the systematic identification of OS risk factors through detailed history and physical examination. Key clinical indicators warranting OS assessment include: leukocytospermia or suspected genital tract infection/inflammation (including dysbiosis); clinical varicocele; major modifiable exposures such as smoking, heat, obesity, or environmental toxicants; recurrent IVF/ICSI failure; poor embryo development; recurrent pregnancy loss; and unexplained infertility with normal conventional semen parameters [51]. This risk-based approach optimizes test selection and resource utilization.

Standard semen analysis according to World Health Organization (WHO) criteria remains the foundation for evaluating male fertility, providing essential information on sperm concentration, motility, and morphology. However, conventional parameters describe sperm quantity, motility, and morphology but fail to uncover underlying molecular dysfunctions, particularly oxidative damage [53]. Therefore, conventional analysis should be viewed as complementary, rather than a substitute for OS biomarker assessment in appropriate clinical contexts.

Chemiluminescence remains a widely used method for ROS detection, offering high sensitivity through luminol-based assays [31,55]; however, chemiluminescence-based ROS measurements are influenced by several biological and methodological variables, including leukocyte contamination, sperm concentration, and sample preparation [56]. Moreover, it should be recognized that the analogue output from luminometers cannot be readily calibrated (it depends on the intrinsic response characteristics of the photomultipliers used in photon detection), so each laboratory must establish its own thresholds of normality. In our andrological laboratory, seminal ROS is assessed by luminol-dependent chemiluminescence using a Berthold AutoLumat luminometer (Germany). Semen aliquots are incubated with modified human tubal fluid (mHTF) and luminol, and the chemiluminescence emitted by ROS-luminol reactions is recorded as counts per minute (cpm) over a 15 min period. For washed semen analysis, sperm suspensions are processed before luminol addition. The assay primarily detects superoxide anion (O_2_^•–^, hydrogen peroxide (H_2_O_2_), and hydroxyl radicals HO^•^, which may be generated by spermatozoa and leukocytes via pathways involving NADPH oxidase activity, mitochondrial electron leakage, L-amino acid oxidase (LAAO), and nitric oxide synthase (NOS). ROS values were adjusted according to sperm concentration relative to a reference value of 20 × 10^6^ spermatozoa. Reference cutoff values established in a Brazilian population of proven-fertile men showed significant variation by leukocyte status. In samples without significant peroxidase-stained leukocyte contamination (Endtz = 0), the cutoff values were 0.51 × 10^4^ cpm/20 × 10^6^ sperm for neat semen. In contrast, samples containing myeloperoxidase-stained leukocytes (Endtz > 1 × 10^6^/mL) showed substantially higher cutoff values, reaching 1.25 × 10^4^ cpm/20 × 10^6^ sperm in neat semen. These findings reinforce the notion that seminal ROS measurements obtained via luminol chemiluminescence reflect the overall oxidative activity of the seminal suspension and may be significantly influenced by leukocyte-derived ROS. Although this methodology still has the limitation of not measuring strictly activated leukocytes, it has proven very helpful in identifying semen samples with elevated ROS, guiding clinical treatment and appropriate individual antioxidant dosing. Further improvements to the protocol using opsonized zymosan can specifically trigger a chemiluminescent response from leukocytes in the presence of luminol and horseradish peroxidase, providing greater accuracy and potentially new avenues for customized treatment. Consequently, ROS thresholds can be interpreted together with leukocyte evaluation and conventional semen parameters, including sperm concentration and motility [31].

Flow cytometry offers greater specificity by distinguishing spermatozoa from leukocytes [57,58] and enabling intracellular ROS measurement with probes such as MitoSOX, though its clinical application is limited by cost and technical complexity. Oxidation-Reduction Potential (ORP) measurement has been proposed as a simplified clinical tool with the MiOXSYS system [59]. However, the way this test is applied has been repeatedly criticized, and we still lack compelling evidence for its clinical utility [60,61].

Total antioxidant capacity (TAC) offers an indirect but clinically relevant measure of redox balance [62,63,64,65], consistently correlating with semen quality and benefiting from recent advances in point-of-care testing [66]. Lipid Peroxidation Biomarkers, such as MDA and isoprostanes, represent specific markers of oxidative membrane damage with demonstrated clinical correlations. Moreover, DNA Oxidation Markers, such as the 8-OHdG measurement, detect oxidative DNA damage and associate it with multiple clinical and lifestyle factors affecting fertility [51,67,68]. Table 2 summarizes a comparison of methods used to assess oxidative stress and antioxidant status in male infertility.

SDF testing quantifies downstream genomic damage resulting from oxidative and other insults. SDF is particularly informative in couples with recurrent pregnancy loss and in unexplained infertility with normal semen parameters. Available platforms include the Sperm Chromatin Structure Assay (SCSA), terminal deoxynucleotidyl transferase dUTP nick-end labeling (TUNEL), alkaline/neutral Comet assay, and sperm chromatin dispersion test. While these tests are heterogeneous and most lack complete standardization and validation, they provide valuable complementary information to direct OS assessment [51].

As an integrated testing strategy, it is pivotal to understand that OS testing alone is most relevant in men with leukocytospermia, suspected infection/inflammation, major modifiable exposures, and for early monitoring after treatment. SDF testing alone is particularly informative in recurrent pregnancy loss and unexplained infertility with normal parameters. Combined OS and SDF testing is recommended in clinical varicocele, repeated IVF/ICSI failure, poor embryo development, and follow-up after targeted therapy [51].

#### 5.2.3. Barriers to Clinical Implementation of OS Based Testing and Future Directions

A main reason for not using OS measurement as a clinical trial selection criterion is the perceived complexity, lack of standardization, and insufficient validation of relevant assays [69]. The lack of standardized, accessible testing methods has limited their routine clinical use [6]. Current priorities include assay standardization, etiologic attribution of DNA damage, and clinical trials testing OS/SDF-guided diagnostic pathways with live birth as the primary endpoint [51]. Direct head-to-head comparisons of TAC methodologies with other diagnostic methods should now be conducted in well-designed clinical trials to accurately evaluate oxidative stress and optimize patient selection for antioxidant treatment. Integration of redox biomarkers with omics technologies and artificial intelligence-driven algorithms may further refine diagnostic precision and enable truly personalized therapeutic interventions [53].

**Table 2:** Comparison of methods used for the assessment of oxidative stress and antioxidant status in male infertility.

Measurement	Assay/Method	Analytical Principle	Target Analyte(s)	Sample Type/Compartment	Notes
**Direct**	Chemiluminescence [55,70]	Detection of photon emission resulting from ROS–probe reactions. Probe/Technique: Luminol.	Target analytes: H_2_O_2_, O_2_^•−^, and HO^•^ (can be improved by using HRP to target H_2_O_2)_	Intracellular and extracellular detection.	**Advantages** High sensitivity; direct ROS measurement; presents minimal cellular toxicity; considered a reference method, provided standardization with and without leukocytospermia **Limitations** High cost; specialized expertise required; inter-laboratory variability; limited cell specificity; interference from leukocyte contamination. **Clinical applications** Oxidative stress diagnosis and monitoring therapy regimens with antioxidants in the treatment of male infertility.
	Flow cytometry [58,71]	Laser-based detection of intracellular ROS and sperm functional parameters using fluorescent probes.	Intracellular ROS (H_2_O_2_, O_2_^•−^, peroxynitrite) and sperm functional biomarkers.	Intracellular	**Advantages** Multiparametric analysis; intracellular ROS detection; cell-specific assessment. **Limitations** High cost; protocol variability; need for specialized instrumentation and trained personnel. **Clinical applications** Advanced diagnostic and research applications.
**Indirect**	Spectrophotometric methods [71]	Quantification of ROS or oxidative biomarkers through colorimetric reactions and absorbance measurements.	ROS and oxidative stress biomarkers (MDA, protein carbonyls, GSH, AOPP, vitamin C).	Mainly extracellular.	**Advantages** Accessible, inexpensive, versatile, and suitable for routine laboratories. **Limitations** Low cost; limited specificity; interference by secondary reactions. **Clinical applications** Routine evaluation of seminal oxidative status and antioxidant interventions.
	TAC [62,64,65]	Assessment of the total antioxidant capacity of biological samples.	Total antioxidant capacity (enzymatic and non-enzymatic antioxidants).	Extracellular detection.	**Advantages** Simple assay; provides global antioxidant balance and status. **Limitations** Low-moderate cost; Indirect assessment of oxidative stress; poor standardization. **Clinical applications** Assessment of antioxidant defenses.
	8-OHdG [51,67]	Measurement of oxidative DNA damage through quantification of 8-OHdG.	Oxidative damage to sperm DNA.	Intracellular detection.	**Advantages** Specific marker of oxidative DNA damage is clinically relevant. **Limitations** High cost; limited availability; lack of assay harmonization. **Clinical applications** Useful in unexplained infertility, recurrent pregnancy loss, and ART failure.

Legends: HRP: horseradish peroxidase, TAC, total antioxidant capacity; 8-OHdG, 8-hydroxy-2′-deoxyguanosine; MDA, malondialdehyde; GSH, reduced glutathione; AOPP, advanced oxidation protein products; ROS, reactive oxygen species.

## 6. The Role of the Andrology Laboratory

Comprehensive, quality-controlled semen analysis provides reliable baseline data for assessing reproductive tract patency and fundamental sperm parameters. However, it is vital to note that semen analysis alone cannot determine fertility, as approximately 25% to 40% of infertile men present with semen parameters within normal reference ranges and are classified as having unexplained infertility. Therefore, semen analysis should be complemented with sperm functional tests. Structural and functional evaluations of spermatozoa, including assessments of ROS, sperm vitality, LPO, DNA fragmentation, 8-OHdG, CK activity, and detection of anti-sperm antibodies, are highly recommended to understand sperm function and fertilization potential. These tests provide crucial information for individualized diagnosis, individualized treatment regimens, and appropriate follow-up, particularly when customized, dose-dependent treatments are implemented, such as selected low-dose, high-quality antioxidants (AOx) [50,72].

Given the clinical importance of detecting white blood cells (WBCs) and the pathological significance of leukocytospermia in infertile patients with elevated OS, the andrology laboratory should routinely conduct quality-controlled leukocyte testing in ejaculate samples. The most widely available and simple method is the Endtz test (myeloperoxidase staining), though it has important limitations [73,74,75]. In this procedure, a small aliquot of liquefied semen is placed in a microtube, mixed with phosphate-buffered saline and a peroxidase substrate solution, and a few microliters of the mixture are examined for brown-stained cells, which indicate the presence of peroxidase-positive granulocytes. [73,74,76].

However, the Endtz test systematically underestimates total leukocyte concentration because activated leukocytes discharge their granules and become peroxidase-negative [77,78]. Since most leukocytes in semen are activated during inflammatory processes, this limitation is clinically significant [75,77]. Studies comparing the peroxidase method with immunocytochemical techniques demonstrate that the peroxidase test has only 58.8% sensitivity when using the WHO threshold of 1 × 10^6^ cells/mL, missing nearly half of leukocytospermic cases [79].

Immunocytochemistry using monoclonal antibodies against pan-leukocyte markers (anti-CD45) or specific leukocyte subpopulations (anti-CD15, anti-CD68) represents a more accurate method for detecting all WBC populations in semen [73,75,79,80,81]. Flow cytometry, combined with anti-CD45 or anti-CD53 antibodies, reliably assesses total leukocyte numbers and differentiates subpopulations, even at low concentrations [80,82]. While immunocytochemical methods are considered the gold standard, they are more expensive, time-consuming, and technically complex, limiting their integration into routine clinical practice [73,75].

Measurement of seminal plasma elastase provides an alternative approach that reflects both granulocyte numbers and their inflammatory activation status [83,84,85]. Polymorphonuclear (PMN) elastase levels above 290–1000 ng/mL correlate well with leukocytospermia (r = 0.755) and can detect “silent” genital tract inflammation even in the absence of elevated leukocyte counts [83,84,85]. Elastase measurement is particularly useful for identifying subclinical inflammation, as 25% of men without leukocytospermia exhibit elevated elastase levels [84]. However, commercial elastase immunoassays are expensive and may yield delayed results, limiting their utility for early diagnosis [74].

Chemiluminescence assays offer a functional approach to leukocyte detection by measuring ROS production following stimulation with leukocyte activators such as opsonized zymosan or the chemotactic peptide N-formyl-methionyl-leucyl-phenylalanine (FMLP) [86,87,88]. When leukocytes are stimulated with FMLP or opsonized zymosan, they produce a burst of ROS that can be quantified through luminol-dependent or lucigenin-dependent chemiluminescence [86,88,89,90]. This method specifically detects functional leukocytes capable of oxidative metabolism and can identify contaminating leukocytes in washed sperm preparations [86]. The FMLP-induced chemiluminescence response is rapid (lag period < 19 s) and specific to leukocytes, as human spermatozoa lack detectable FMLP receptors [86,87]. While promising, chemiluminescence testing requires specialized equipment and has not been widely adopted in routine clinical andrology laboratories.

Male genital tract infections primarily generate extracellular ROS, which, together with inflammation, chemotaxis, and leukocyte activation, initiate a self-perpetuating cascade of the myeloperoxidase system, producing additional ROS to combat the infectious agent [91]. This inflammatory response can cause oxidative damage to spermatozoa, impairing motility, morphology, acrosome function, and increasing DNA fragmentation [73]. Given the clinical importance of leukocyte infiltration, it is disconcerting that considerable uncertainty persists over the definition of pathological leukocytospermia and the relationship between WBCs and seminal oxidative stress [73]. The WHO defines pathological leukocytospermia as more than 1 × 10^6^ WBC/mL in semen. However, the minimum WBC count that impairs fertility remains undetermined. When treating infertile men with detectable leukocytes in the ejaculate, any amount should be carefully evaluated prior to initiating AOx therapy. In a study comparing OS in semen samples with varying leukocyte counts (measured by the Endtz test) following a wash-and-resuspend procedure, oxidative stress was assessed using the ROS-TAC score, a previously validated composite index reflecting the balance between total antioxidant capacity (TAC) and reactive oxygen species (ROS). Lower ROS-TAC scores indicate greater antioxidant protection against oxidative stress, whereas higher scores indicate greater oxidative stress. Samples without seminal leukocytes exhibited significantly lower ROS levels and lower ROS-TAC scores compared to samples containing any seminal leukocytes, even at very low concentrations. The observations indicate that oxidative stress occurs even in patients with very low seminal WBC counts (0–1 × 10^6^/mL) and increases with higher WBC counts [92].

## 7. The Significance of Specific Micronutrients and Vitamins, as Well as Their Antioxidant and Pro-Oxidant Properties

### 7.1. Criteria for Selecting Antioxidants in the Management of Male Infertility

Given the extensive range of micronutrients and antioxidants, including natural, synthetic, and plant-derived compounds, available from both established industries and compounding pharmacies, a comprehensive review is beyond the scope of this manuscript. The eligibility criteria for inclusion as adjunctive therapy for male infertility are as follows: oral bioavailability; presence in the seminal fluid of normozoospermic fertile men and deficiency or absence in infertile men; demonstrated efficacy in animal or, preferentially, in human studies using validated quality-controlled standardized assays for OS, ROS, LPO, and DNA damage; avoidance of supraphysiological dosages; high-quality non-synthetic components must act synergistically; exclusion of substances with antagonistic or neutralizing interactions or those that, in combination, may synergistically exacerbate oxidative conditions, induce reductive stress, or exacerbate the ROS-TAC ratio. Micronutrients that induce cross-chemical reactions with physiologically available transition metals, or that trigger metal-induced reactions upon external administration, should also be avoided, even at low to moderate doses, as such interactions may enhance adverse effects or cause cellular damage. Attention should be given to other medications often prescribed for concomitant medical conditions, which may contain components that interact with the proposed antioxidant therapy and trigger cross-reactions that initiate the Fenton and Haber-Weiss reactions.

### 7.2. Clinical Management of Oxidative Stress: Integrating Translational Science with Medical Practice

**Figure 2:**
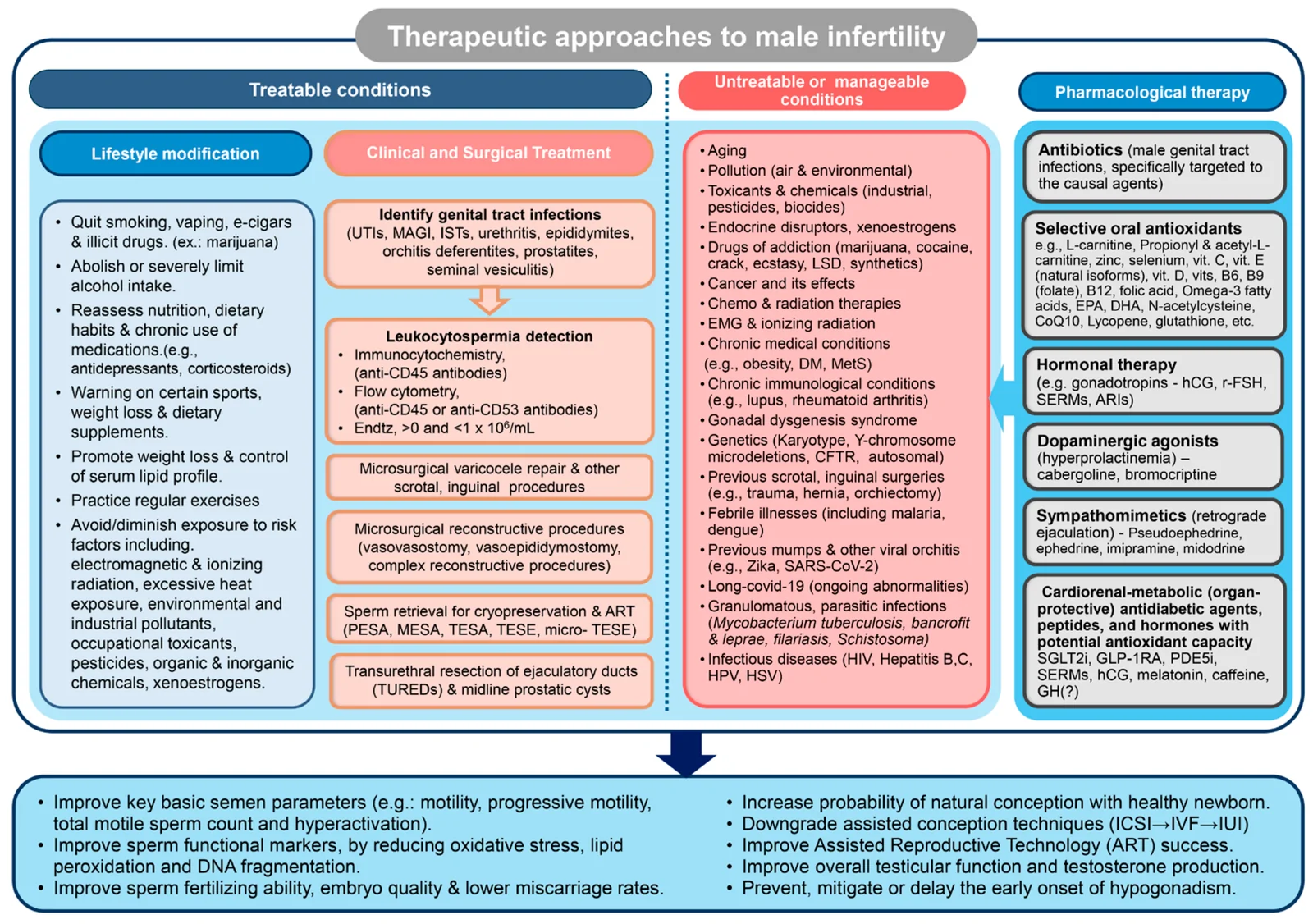
A comprehensive, broad overview of major therapeutic approaches to male infertility. Pharmacological therapy includes a wide variety of medications, hormones, supplements, nutraceuticals, and drugs with potential antioxidant metabolic effects that, individually or used in combination in a clinical and laboratory-based setting, using individualized precise medicine, may provide better results than a random or “one-size-fits-all” approach for oxidative stress-related spermatozoa and testicular dysfunction in men presenting with subfertility or infertility. Management should integrate the identification of treatable and non-treatable conditions, lifestyle modification, targeted clinical or surgical interventions, and individualized pharmacological therapy. This multimodal approach aims to reduce oxidative stress, improve testicular and sperm function, and semen quality. Furthermore, optimize reproductive outcomes by fostering natural pregnancy, reducing ART complexity, and improving success rates.

The proposed approach to improving fertility status in male infertility through antioxidant adjuvant therapy involves a comprehensive assessment of patient clinical characteristics, including scrotal and testicular conditions (e.g., varicocele, orchitis, epididymitis), comorbidities, chronic diseases, and prior chemotherapy or radiation therapy. A key aspect of antioxidant treatment regimens is understanding whether the underlying pathology driving testicular and sperm oxidative stress is congenital, genetic, or acquired. For example, cryptorchidism, ectopic testis, and gonadal dysgenesis syndrome are clearly of embryological origins, with anatomical, physiological, and functional abnormalities in the testis since the early fetal period, and therefore less likely to respond or to maintain eventual sperm function improvement for a long time. On the other hand, if the condition is acquired and surgically or medically treatable (e.g., varicocele, vasectomy reversal, infections, obesity, diabetes, dyslipidemia, metabolic conditions, SARS-CoV-2 infection, substance abuse), the improvement is likely to last or at least be maintained for a sufficient time to achieve natural pregnancy, generate sufficient spermatozoa for cryopreservation, and facilitate the use of medically assisted reproduction. Therefore, it is crucial to determine precisely whether the origins of OS are attributable to treatable conditions (vide supra) or to non-treatable factors (such as aging, prior chemotherapy, radiation, environmental toxins, and long COVID-19). A proposed translational framework for individualized management of oxidative stress–related male infertility is summarized in Figure 2.

Consideration must be given to habits and lifestyle factors such as smoking, alcohol and drug use, and nutritional status, as well as chronic environmental exposures to high levels of air pollution and constant exposure to electromagnetic radiation, even though establishing a cause-and-effect correlation is often difficult. Therefore, diagnoses commonly associated with male infertility, such as varicocele and sexually transmitted infections (including Chlamydia, Ureaplasma, Mycoplasma, and gonorrhea), along with clinical or subclinical infections and inflammations of the reproductive tract (e.g., urethritis, epididymitis, prostatitis), and testicular or epididymal abnormalities, should be diagnosed and addressed as the primary source of OS or a contributor in conjunction with another significant cause (e.g., obesity, varicocele, SARS-CoV-2 infection, cannabis, etc.). These assessments should be aligned with quality-controlled semen analysis, including sperm functional testing, like the measurement of ROS, LPO, DNA fragmentation, and CK when sperm immaturity is present in the semen analysis or if a varicocele is diagnosed, both at baseline and during treatment follow-up, to tailor antioxidant therapy intensity to the level of OS. In conducting these analyses, reductions should be sought in ROS generation, LPO, and DNA fragmentation levels, as measured by standardized, quality-controlled methods, preferably familiar to each andrology laboratory and population-specific [31]. It is essential to assess OS levels in semen before determining the most appropriate therapeutic strategy. Additionally, leukocytospermia should be identified and managed prior to or during antioxidant therapy. The new WHO 6th edition laboratory manual for the examination and processing of human semen now includes oxidative stress testing as an advanced complementary test following standard semen analysis. This inclusion supports routine OS testing in advanced andrology laboratories, beyond research-only purposes, facilitating a more efficient, reliable, and outcome-oriented approach to improving semen quality [4].

Experimental models have demonstrated the dual effects of AOx supplementation on male reproductive health when administered without prior assessment of redox status [50,93,94]. Such studies underscore the potential risks associated with high doses of commonly prescribed, compounded, or over-the-counter micronutrients [50,95]. In a dose-escalation study using healthy CD1 mice and subfertile, oxidatively stressed Gpx5^−/−^ mice, the effects of common water-soluble antioxidant micronutrients (vitamin C, zinc, folate, and carnitine) on semen parameters, systemic redox balance, sperm DNA structural integrity, and fertility outcomes were evaluated over one spermatogenic cycle [50]. In healthy mice with normal redox balance, increasing doses of individual micronutrients produced minimal changes in semen parameters. However, combined high doses of vitamin C, zinc, folate, and carnitine significantly disrupted redox balance in blood plasma and compromised sperm DNA integrity in an ingredient-specific manner [50]. Notably, moderate-to-high doses of carnitine resulted in severe, dose-dependent DNA fragmentation, as assessed by the TUNEL assay, a finding confirmed in a subsequent experiment with the highest carnitine dose. In this follow-up experiment, healthy male mice supplemented with carnitine and mated to females exhibited reduced pregnancy rates and fewer total pups. Conversely, in oxidatively stressed Gpx5^−/−^ mice, high doses of carnitine had the opposite effect, improving sperm DNA integrity [50].

These findings are consistent with the broader concept of the “antioxidant paradox” in male infertility, whereby excessive antioxidant intake can induce reductive stress—a state as deleterious to sperm function as oxidative stress itself [93,96,97]. Reductive stress has been shown to interfere with essential ROS-dependent signaling pathways required for sperm capacitation, hyperactivation, and acrosome reaction [95,98]. Furthermore, recent evidence suggests that antioxidant supplementation in healthy males may alter sperm DNA methylation and hydroxymethylation patterns, raising concerns about paternally inherited epigenetic alterations [99,100].

These observations indicate that, despite strong evidence supporting preconception antioxidant use to enhance semen quality in men with documented oxidative stress, reproductive specialists should base clinical decisions on oxidative stress assessment, patients’ comorbidities, cofactors, and recognized pathologies such as varicocele or exposure to environmental pollutants and recommend individualized antioxidant dosing [17,94,97]. This approach aims to improve semen quality, prevent reductive stress, avoid deterioration of sperm and testicular function, and reduce the risk of adverse reproductive outcomes [50,95]. Importantly, the MOXI randomized clinical trial demonstrated that a combined antioxidant formulation (vitamin C, vitamin E, selenium, L-carnitine, zinc, folic acid, and lycopene) did not improve semen parameters, DNA fragmentation, or live-birth rates in men with male factor infertility. However, because this study failed to select patients based on oxidative stress, it underscored the need to diagnose seminal redox status as a prerequisite for initiating antioxidant therapy [101].

## 8. Mechanism-Based Antioxidant Therapy of Oxidatively Induced Sperm Damage

### 8.1. Oxidative Stress: A Convergent Mechanism of Male Reproductive Dysfunction

Although multiple factors contribute to male infertility, OS is among the few well-established causes of male reproductive dysfunction. Substantial evidence accumulated over recent decades demonstrates that oxidative stress not only correlates with impaired sperm function but also actively contributes to its development. This distinction is significant, as most traditional diagnostic classifications in andrology rely on clinical findings or semen abnormalities, whereas oxidative stress is a biological mechanism that can directly diminish male fertility [6].

Reactive oxygen species are central to this process; however, ROS are not inherently detrimental. At physiological concentrations, ROS facilitate essential aspects of sperm function, including capacitation, hyperactivation, the acrosome reaction, and sperm–oocyte interaction. Thus, redox signaling is critical for normal fertilization. Male reproductive dysfunction arises when excessive ROS production exceeds local antioxidant defenses, disrupting redox balance. Under these conditions, normal redox regulation is disrupted, leading to harmful OS [102].

This concept holds clinical significance, as a wide range of conditions can induce oxidative stress. Disorders of the male genital tract, systemic metabolic diseases, aging, environmental exposures, and unhealthy lifestyle habits have all been associated with either excessive ROS production or insufficient antioxidant defenses. Despite their diverse etiologies and clinical manifestations, these conditions frequently converge on a common state of redox imbalance. Consequently, oxidative stress serves as a unifying mechanism underlying multiple, seemingly unrelated, causes of male infertility [98,103,104].

The effects of oxidative stress are consistent regardless of the initiating factor. Excessive ROS induces a characteristic pattern of cellular damage, impacting lipids, mitochondria, DNA, and proteins, and activating cell death pathways. Although the extent of these alterations may vary among individuals, the trajectory of sperm damage remains similar. Therefore, oxidative stress is more accurately regarded as a common consequence of various diseases rather than as an independent disease entity [6].

Acknowledging oxidative stress as a common factor in male reproductive dysfunction necessitates a shift in therapeutic strategies. If numerous causes of infertility primarily disrupt redox balance, interventions should prioritize identifying and addressing the underlying sources of oxidative injury. This perspective underpins mechanism-based antioxidant therapy, which seeks to employ antioxidants with greater specificity and mechanistic insight, rather than relying exclusively on general supplementation [105].

### 8.2. Clinical Conditions Associated with Oxidative Stress

After oxidative stress is established as a central mechanism in male reproductive dysfunction, it becomes essential to identify the various clinical conditions that can induce it. Oxidative stress is not confined to a single disease but may arise from a wide range of pathological, environmental, and physiological factors. Although these conditions differ in etiology and clinical presentation, all disrupt redox homeostasis by generating excess ROS, impairing antioxidant defenses, or both. Consequently, oxidative stress serves as a common link among otherwise unrelated causes of male infertility. It is estimated to contribute to 25% to 87% of male subfertility cases, highlighting its extensive impact and clinical significance in male infertility [17,102,106].

Multiple disorders of the male reproductive tract, such as varicocele, genital tract infections and inflammation, are consistently associated with elevated oxidative stress. These conditions promote ROS generation through distinct mechanisms: tissue hypoxia and impaired venous drainage in varicocele; leukocyte activation and inflammatory cytokine release during infections and inflammation; and direct interactions between pathogens and spermatozoa in certain infections [104,107,108]. Despite differing initiating factors, the resultant redox imbalance creates an environment that facilitates sperm dysfunction. In varicocele, hemodynamic disturbances, hyperthermia, hypoxia, inflammation, and oxidative stress interact to perpetuate testicular injury [108,109,110]. Infections and inflammation in the male genital tract frequently induce leukocyte recruitment and activation, resulting in increased ROS production and oxidative damage [73,104,107,111].

Oxidative stress may also originate from systemic disorders outside the reproductive tract. In metabolic conditions such as obesity, metabolic syndrome, and type 2 diabetes mellitus, chronic low-grade inflammation elevates ROS production, while mitochondrial dysfunction disrupts cellular energy homeostasis. Altered endocrine signaling modulates hormone levels that regulate antioxidant enzymes, and diminished antioxidant capacity reduces the ability to neutralize oxidative molecules [38,98,112,113]. These abnormalities contribute to systemic oxidative stress, which can subsequently impair testicular function, spermatogenesis, and sperm quality. The reproductive consequences of these disorders arise not only from hormonal disturbances but also from persistent redox imbalance. This underscores the strong association between systemic metabolic health and male reproductive potential [98,102].

Lifestyle-related factors represent significant sources of oxidative stress in the male reproductive system. Behaviors such as smoking, excessive alcohol consumption, sedentary lifestyle, psychological stress, poor diet, and obesity are associated with increased ROS production and diminished antioxidant defenses [36,37,39,102,113]. Environmental exposures, including air pollution, heavy metals, endocrine disruptors, pesticides, bisphenols, phthalates, and microplastics, further elevate this risk [23,24,113]. These factors commonly induce oxidative injury by enhancing ROS generation, impairing mitochondrial function, and reducing antioxidant reserves [98]. The widespread prevalence of these contributors accounts for the frequent occurrence of OS in infertile males. Notably, risk factors often coexist, including obesity, physical inactivity, environmental exposure, aging, and subclinical inflammation, which collectively amplify OS and impair sperm function. This complex etiology complicates the identification of a single causative factor and highlights the importance of focusing on redox imbalance rather than solely on disease classification [114].

Aging also contributes to oxidative stress and the decline of male reproductive function. Advancing paternal age is associated with increased mitochondrial dysfunction, resulting in the accumulation of oxidative damage, reduced antioxidant efficiency, and a heightened risk of DNA damage [6,98,115]. Although aging is not classified as a disease, age-related alterations in redox regulation may promote oxidative injury and exacerbate the effects of other risk factors. Therefore, oxidative stress is likely a principal pathway connecting aging to reduced male reproductive capacity.

Collectively, these observations demonstrate that oxidative stress is not confined to a single clinical condition. Rather, it emerges as a biological response to a spectrum of reproductive, systemic, environmental, lifestyle-related, and age-associated challenges [102]. The convergence of these diverse factors on redox imbalance accounts for the pervasive influence of oxidative stress in male infertility. Importantly, this understanding challenges the traditional approach of classifying patients solely by disease category, as disparate clinical conditions can result in similar oxidative injury. Consequently, the management of oxidative stress-related male infertility should shift from a disease-based to a mechanism-based diagnostic framework. In this paradigm, the identification of oxidative damage, redox disturbances, and critical pathways of sperm injury becomes central to clinical decision-making [6]. Elucidating the molecular pathways underlying oxidative insults is essential for understanding the mechanisms of sperm dysfunction.

### 8.3. Pathophysiology of Oxidatively Induced Sperm Damage

Oxidative stress impairs male fertility through conserved molecular events that compromise sperm function and reproductive potential, regardless of the underlying cause. Human spermatozoa are particularly susceptible to oxidative injury because adaptations for fertilizing ability limit their defense and repair mechanisms. During spermiogenesis, spermatozoa lose most of their cytoplasm, including antioxidant systems and DNA repair capacity. Their membranes, enriched in polyunsaturated fatty acids (PUFAs), are highly vulnerable to oxidative attack. Sperm generates ROS via mitochondrial activity and specialized oxidase systems. When ROS production surpasses physiological thresholds, sperm rapidly incur injuries affecting membranes, mitochondrial function, signaling pathways, and genomic stability [6,18,102,116].

Lipid peroxidation is among the earliest consequences of OS in sperm. While the high PUFA content of sperm membranes is essential for membrane fluidity and fusion during fertilization, it also renders sperm highly susceptible to free radical attack. ROS-induced hydrogen abstraction from membrane lipids initiates a chain reaction that generates lipid hydroperoxides and reactive electrophilic aldehydes, particularly malondialdehyde (MDA) and 4-hydroxynonenal (4-HNE) [15,18,102]. These aldehydes are not merely markers of injury; they actively induce cellular dysfunction. Through covalent modification of proteins, phospholipids, and nucleic acids, 4-HNE exacerbates damage throughout the sperm cell. This leads to reduced membrane fluidity, decreased motility, impaired capacitation, diminished acrosomal response, and compromised sperm–oocyte interaction. Furthermore, 4-HNE can stimulate mitochondrial ROS production and apoptotic signaling, propagating injury beyond the plasma membrane and perpetuating the oxidative cascade [6,42,117].

Mitochondria play a central role in the pathological cascade of oxidative stress, functioning as both sources and targets of ROS. Under physiological conditions, mitochondrial ROS supports redox signaling necessary for sperm function. However, excessive ROS impairs the electron transport chain, disrupts membrane potential, and compromises oxidative phosphorylation. Electrophilic aldehydes generated by lipid peroxidation, particularly 4-HNE, form adducts with mitochondrial proteins, such as succinate dehydrogenase, which promote electron leakage and further ROS production [42]. This establishes a self-amplifying ROS cycle in which oxidative damage perpetuates further injury. Reduced ATP production diminishes sperm motility and viability, while ongoing mitochondrial dysfunction increases the overall cellular oxidative burden [18,42,118]. In addition to mitochondrial sources, recent evidence suggests that enzymatic ROS generators, including NOX5 and IL4I1, also contribute to oxidative stress in human sperm, highlighting the complexity of sperm redox biology [6]. As oxidative injury advances, it affects the sperm genome. Oxidative DNA damage represents a significant clinical consequence of excessive ROS exposure, influencing fertilization, embryo development, pregnancy outcomes, and offspring health [119]. ROS induce DNA damage by oxidizing nucleotide bases and abstracting hydrogen from the deoxyribose backbone, resulting in oxidized adducts and strand breaks. Among these lesions, 8-OHdG serves as a hallmark biomarker of oxidative DNA damage and is strongly associated with sperm DNA fragmentation [119]. Susceptibility to oxidative DNA damage varies among spermatozoa. According to the two-step hypothesis proposed by Aitken and colleagues, defective spermiogenesis results in spermatozoa that are poorly protaminated, exhibit abnormal chromatin remodeling, retain residual cytoplasm, have increased histone content, and are impaired in maturation. These dysmature sperm cells are primary targets for oxidative attack, often originating from their own dysfunctional mitochondria, leading to the accumulation of oxidative lesions and DNA fragmentation [119,120]. Because mature spermatozoa have limited DNA repair capacity, oxidative lesions persist until fertilization, at which point repair depends on the oocyte.

Controlled ROS generation modulates cyclic AMP production, protein phosphorylation, cholesterol efflux, and membrane remodeling, all of which contribute to fertilizing competence. When ROS levels exceed physiological thresholds, these signaling pathways become dysregulated. Consequently, oxidative stress impairs sperm function by inducing molecular damage and disrupting redox-dependent signaling required for fertilization. This dual role of ROS as both essential signaling molecules and potential mediators of injury constitutes the oxidative paradox in male reproductive biology [6,116,117].

Persistent OS can activate regulated cell death pathways in sperm. Oxidative injury may initiate apoptosis-like signaling specific to sperm, characterized by mitochondrial ROS generation, phosphatidylserine externalization, caspase activation, and cellular senescence [22,119]. Recent studies indicate that alternative forms of regulated cell death, such as autophagy-associated cell death and ferroptosis, also contribute to male reproductive dysfunction. Ferroptosis is particularly significant because it is driven by iron-dependent lipid peroxidation, directly linking it to the oxidative environment present in many cases of male infertility [48,103]. Although these pathways differ mechanistically, all are triggered by oxidative stress and exemplify the diverse cellular outcomes resulting from redox imbalance.

Lipid peroxidation, mitochondrial dysfunction, oxidative DNA damage, altered redox signaling, and regulated cell death may appear as distinct phenomena, yet they frequently co-occur as interconnected outcomes of a shared oxidative cascade. The clinical presentation in infertile men is influenced not only by the presence of oxidative stress but also by the predominant mechanism of sperm injury and the interactions among these pathways. Some patients primarily exhibit membrane dysfunction and reduced motility, while others present with extensive DNA damage, mitochondrial failure, or activation of cell death pathways. This mechanistic diversity explains why individuals with similar clinical diagnoses can display markedly different patterns of sperm dysfunction. It also underscores the importance of mechanism-based diagnostic testing and targeted antioxidant interventions. Such approaches enable more precise and individualized management of oxidative stress-related male infertility. Consequently, antioxidant therapy should be directed toward the primary oxidative mechanisms underlying sperm dysfunction in each patient, rather than relying solely on the overall clinical diagnosis or initial ROS levels.

### 8.4. Clinical Principles of Mechanism-Based Antioxidant Therapy

The increasing use of antioxidant supplementation in male infertility reflects the recognition of oxidative stress as a significant factor impairing sperm function. Despite extensive research and numerous clinical studies, the efficacy of antioxidant therapy remains controversial. Although improvements in semen parameters are frequently observed, robust evidence for significant improvements in reproductive outcomes, such as pregnancy and live birth, remains inconsistent, largely due to methodological flaws in patient selection, risk factor assessment, and the reliable documentation of OS [121,122,123].

The MOXI trial, one of the largest randomized placebo-controlled studies, exemplifies this uncertainty. The trial, which used a combination of common antioxidants, did not demonstrate significant improvements in semen parameters, sperm DNA fragmentation, pregnancy rates, or live birth rates compared with placebo [101]. These findings highlight those key limitations in many antioxidant trials, including heterogeneous populations, inadequate duration, and insufficient mechanistic characterization or confirmation of oxidative stress, which may contribute to ambiguous outcomes.

These findings reveal a primary limitation: traditional empirical antioxidant therapy frequently lacks objective evidence of oxidative stress in selected patients. Because OS represents a biological mechanism rather than a clinical diagnosis, clinical management should begin with a mechanism-based assessment. Employing validated biomarkers, such as ROS measurement, ORP, MDA, 4-HNE, 8-OHdG, or SDF, to confirm OS can enhance patient selection and reduce unnecessary antioxidant use [6,102].

A second core principle is that antioxidant supplementation should not replace efforts to identify and address the underlying cause of oxidative stress. Treating the primary condition, such as a varicocele, infection, obesity, or adverse lifestyle factors, often yields greater benefits than antioxidant therapy alone [98,102]. Evidence suggests that targeted interventions, including varicocelectomy or lifestyle modifications, can improve outcomes more effectively than antioxidants administered in isolation and without criteria [110]. Therefore, antioxidants should be regarded as adjuncts rather than substitutes for primary treatments.

Oxidative stress-related male infertility encompasses diverse mechanisms, indicating that not all patients benefit equally from identical antioxidant regimens. Treatments should be tailored to the specific intracellular organelles affected, considering the endpoints of injury mechanisms in a well-established clinical diagnostic scenario. For instance, classify the endpoint as mitochondrial dysfunction, lipid peroxidation, or DNA oxidation with or without relevant DNA fragmentation. Although supporting evidence continues to develop, a mechanism-based approach is more rational and potentially more effective than generalized random antioxidant therapy [6].

Avoiding overtreatment is critically essential. Not all ROS are detrimental; some are necessary for critical sperm functions. Excessive antioxidant use may disrupt physiological processes and induce reductive stress, highlighting the importance of rational dosing and careful selection. Furthermore, antioxidant therapy carries potential risks and should not be considered universally benign [124].

Antioxidant therapy requires objective monitoring and timely adjustment. Instead of relying on prolonged empirical regimens, patients should undergo periodic assessments, including semen analysis, SDF, and, preferentially, redox biomarker evaluation. This approach ensures that treatment remains aligned with the underlying injury mechanism and facilitates timely modification or discontinuation of therapy [121]. Thus, Table 3 summarizes the mechanisms of action, potential benefits, and possible risks of antioxidants and micronutrients currently used in male infertility.

Cardiorenal-metabolic therapies have emerged as promising adjuncts in the management of oxidative stress–related male infertility, particularly in patients with obesity and type 2 diabetes mellitus. Beyond their established cardiovascular and renal protective effects, glucagon-like peptide-1 receptor agonists (GLP-1RAs) and sodium-glucose cotransporter-2 inhibitors (SGLT2is) appear to modulate testicular redox homeostasis through complementary mechanisms. GLP-1RAs improve mitochondrial function, reduce ROS generation, enhance insulin sensitivity, and support Leydig cell steroidogenesis, whereas SGLT2is attenuate glucotoxicity and activate endogenous antioxidant pathways, including the nuclear factor erythroid 2-related factor 2/heme oxygenase-1 (Nrf2/HO-1) signaling axis, resulting in reduced lipid peroxidation, preservation of testicular architecture, and decreased germ cell apoptosis [144,145,146,147]. Additional compounds, including melatonin, phosphodiesterase type 5 inhibitors, and selective estrogen receptor modulators, have also demonstrated antioxidant and redox-modulating properties in experimental and clinical settings, although evidence supporting their use as targeted therapies for oxidative stress–induced male infertility remains limited [148,149,150]. Collectively, these agents represent a new generation of organ-protective therapies with potential reproductive benefits, warranting further investigation in prospective clinical trials incorporating reproductive outcomes and validated oxidative stress biomarkers.

In summary, future antioxidant therapy for male infertility should avoid indiscriminate application and be individualized with customized dosing of high-quality ingredients. Advancements depend on the use of redox biomarkers, functional sperm testing, precise mechanism identification, and individualized treatment strategies. Targeted antioxidant therapy should be reserved for carefully selected patients in whom oxidative stress is clearly identified as a predominant factor, taking into account underlying medical and clinical conditions and whether the primary source is treatable [6].

## 9. Conclusions

Oxidative stress represents a highly prevalent and potentially modifiable component contributing to male infertility. Effective management should extend beyond basic semen analysis and empirical antioxidant administration, instead emphasizing mechanism-based diagnosis and individualized, customized dosing and treatment strategies. The evidence presented in this manuscript demonstrates that oxidative stress is both diagnosable and monitorable. Comprehensive andrological evaluation, assessment of redox-related biomarkers, and identification of treatable or modifiable conditions are essential. Antioxidant therapy should be individualized for each patient based on their oxidative status, clinical characteristics, and underlying etiologies. Advancements in this field will require standardized diagnostic protocols, routine implementation of functional sperm assessments, and rigorous translational research. This approach has the potential to improve testicular and sperm function, increasing the likelihood of natural conception, optimizing reproductive outcomes when medically assisted reproduction is strictly necessary, and potentially promoting better offspring health. Furthermore, enhance overall male general health and quality of life, promoting the adoption of precision-guided medicine in the management of male infertility and preventing, mitigating or delaying the early onset of male hypogonadism.

## Figures and Tables

**Table 1:** Antioxidant Defense Components in Human Semen: Non-Enzymatic Molecules and Enzymatic Systems [6,15].

Non-Enzymatic Antioxidants Present in Semen (Seminal Plasma and Sperm Cells)	Enzymes and Proteins with Antioxidant Activity Are Present in Seminal Plasma (and/or Produced by the Testes, Epididymis, and Accessory Glands)
Ascorbic acid (vitamin C) Alpha-tocopherol (vitamin E) Uric acid Reduced glutathione (GSH) and the GSH/GSSG ratio Oxidized glutathione (GSSG) part of the redox system Coenzyme Q10 (ubiquinone/ubiquinol) Taurine and hypotaurine Carnitines (L-carnitine and Acetyl-L-carnitine) Tyrosine and other amino acids with radical-scavenging/chelating capacity Pyruvate Melatonin Retinol/derivatives of vitamin A Bilirubin and other chelating molecules Carrier/binding proteins with antioxidant roles (albumin, binding factors) Dietary/phytonutrient compounds in smaller amounts (flavonoids, polyphenols), depending on diet	Superoxide dismutase (SOD)—mainly Cu/Zn-SOD isoforms in plasma Catalase Glutathione peroxidase (GPx), including GPx4 (PHGPx —important in spermatozoa) Glutathione reductase (GR) Glutathione S-transferase (GST) Peroxiredoxins (PRDX1–6) Thioredoxin/thioredoxin reductase system (Trx/TrxR) Glutaredoxins Paraoxonase (PON) associated with protection against lipid peroxidation in seminal plasma Enzymes involved in the removal/repair of oxidative damage (some specific lipases/esterases)

Notes: Many of these antioxidants are distributed between seminal plasma and cells (spermatozoa, epididymis); some enzymes (e.g., GPx4, PRDX6) are especially important inside the sperm to protect membranes and DNA; Composition and levels vary between individuals, health status, diet, and collection procedure [4,6,15].

**Table 3:** Mechanisms of action, potential benefits, and possible risks of antioxidants and micronutrients used in male infertility.

Antioxidant/Micronutrient	Proposed Mechanisms	Potential Benefits	Possible Risks/Main Limitations
**Vitamin C** [123]	Water-soluble free radical scavenger. Neutralizes free radicals.	↓ Oxidative stress. Possible improvement in sperm DNA integrity.	High doses may cause gastrointestinal discomfort and, in rare cases, nephrolithiasis. Excessive supplementation may disrupt physiological ROS-mediated signaling pathways.
**Vitamin E** [121,125,126]	A major lipid-soluble antioxidant that terminates lipid peroxidation chain reactions and protects cellular membranes against oxidative damage.	Protection of membrane integrity; possible improvement in motility.	Excessive supplementation may increase bleeding risk in susceptible individuals. High-dose vitamin E (≥400 IU/day) has been associated with increased all-cause mortality, while the long-term safety of synthetic vitamin E remains uncertain. Moreover, high doses of α-tocopherol may disrupt redox balance by suppressing ROS, promoting RNS formation, and reducing circulating γ-tocopherol levels.
**Vitamin B6** **(pyridoxine)** [127]	Cofactor for more than a hundred metabolic enzymes; Supports glutathione synthesis, homocysteine metabolism, and ROS scavenging.	Enhances antioxidant defenses and may support sperm function.	Limited clinical evidence; high doses may cause neuropathy and adversely affect semen quality in animal studies.
**Vitamin B9 (Folate, natural form; Folic Acid, synthetic form)** [128,129]	Acts as a coenzyme in one-carbon metabolism, supporting DNA and RNA synthesis, nucleotide biosynthesis, and cellular methylation reactions essential for cell division and genomic stability.	May improve DNA integrity, antioxidant capacity, and cellular metabolism.	Excessive folic acid intake may mask the hematological manifestations of vitamin B12 deficiency, potentially delaying diagnosis while neurological damage progresses.
**Selenium** [122,130]	Cofactor of glutathione peroxidases. Enhances glutathione peroxidase activity and protects against oxidative cellular damage.	↑ Motility, ↑ sperm quality, and ↓ oxidative damage.	Excess intake may cause selenosis (hair loss, nail changes, gastrointestinal symptoms).
**Zinc** [121,131]	Cofactor for antioxidant enzymes; involved in spermatogenesis, chromatin stabilization, and testosterone metabolism.	Potential improvement in sperm concentration and morphology.	Gastrointestinal symptoms, lethargy, hallucinations, and chronic excess may induce copper deficiency. Excess supplementation may impair copper absorption.
**Omega-3 fatty acids (EPA, DHA, and DPA)** [132,133]	Incorporate into sperm plasma membranes, improving membrane fluidity and integrity; modulate inflammatory pathways and exert indirect antioxidant effects by reducing reactive oxygen species production and oxidative stress.	↑ Sperm concentration, improved membrane function, may reduce oxidative damage and inflammation.	Gastrointestinal discomfort (including diarrhea), headache, and fishy body odor have been reported. High doses may increase susceptibility to lipid peroxidation and bleeding in predisposed individuals. Clinical outcomes remain heterogeneous across studies.
**Glutathione** [134]	Major intracellular antioxidant; directly scavenges ROS and serves as a cofactor for glutathione peroxidase.	Protects against oxidative damage, supports redox balance, and may improve cellular function.	Oral bioavailability is variable; clinical efficacy may depend on formulation and dosage. Limited high-quality clinical evidence for some indications.
**NAC** [121,135]	Precursor of glutathione; directly scavenges ROS and enhances endogenous antioxidant systems.	↓ Oxidative stress. Possible improvement in sperm motility, protamine content, and DNA integrity.	Nausea, dyspepsia, headache. Excessive ROS scavenging may interfere with physiological redox signaling.
**L-Carnitine/Acetyl-L-Carnitine/Propionyl-L-carnitine** [50,122,136,137,138]	Facilitates mitochondrial fatty acid transport.	↑ Sperm energy metabolism, ↑ progressive motility, ↑ total motility, and possible improvement in morphology. Propionyl-L-carnitine reduces oxidative stress, leukocyte activation, and erectile dysfunction.	Mild gastrointestinal symptoms and occasional changes in body odor have been reported. L-Carnitine: High-dose supplementation may compromise sperm DNA structural integrity and has been associated with the progression of carotid stenosis. Evidence regarding its clinical efficacy remains inconsistent.
**CoQ10** [139,140,141,142]	Improves mitochondrial function and ATP production.	↑ Sperm concentration, ↑ motility, ↑ seminal antioxidant capacity, ↓ oxidative stress markers. Possible increase in clinical pregnancy rate.	Gastrointestinal discomfort, headache, and nausea. Low oral bioavailability (≈2–3%); prolonged supplementation or specialized formulations may be required.
**Lycopene** [123,143]	Neutralizes free radicals.	Possible improvement in sperm concentration and motility.	Generally, well tolerated. Could cause lycopenemia. Limited evidence from large randomized clinical trials; absorption is influenced by dietary fat intake.

Legends: ROS, reactive oxygen species; RNS, reactive nitrogen species; CoQ10, Coenzyme Q10; NAC, N-Acetylcysteine (NAC), DHA, docosahexaenoic acid; EPA, eicosapentaenoic acid; DPA, docosapentaenoic acid. ↑ increases or increased; ↓ reduces or reduced.

## Data Availability

The original contributions presented in this study are included in the article. Further inquiries can be directed to the corresponding author.

## Abbreviations

The following abbreviations are used in this manuscript:

8-OHdG	8-hydroxy-2-deoxyguanosine
ABTS	2,2′-azino-bis (3-ethylbenzothiazoline-6-sulfonic acid)
AOx	Antioxidants
ART	Assisted Reproductive Technology
ARIs	Aromatase Inhibitors
AUC	Area Under the Curve
BMI	Body Mass Index
CK	Creatine Kinase
cpm	Counts Per Minute
CoQ10	Coenzyme Q10
COVID-19	Coronavirus Disease 2019
CFTR	Cystic Fibrosis Transmembrane Conductance Regulator
DCFH-DA	2′,7′-Dichlorodihydrofluorescein Diacetate
DM	Diabetes Mellitus
DHE	Dihydroethidium
DNA	Deoxyribonucleic Acid
DHA	Docosahexaenoic acid
EPA	Eicosapentaenoic acid
EMG	Electromagnetic radiation
G-6-PDH	Glucose-6-Phosphate Dehydrogenase
GSH	Glutathione
GH	Growth Hormone
GLP-1RA	Glucagon-Like Peptide-1 Receptor Agonists
H_2_O_2_	Hydrogen Peroxide
hCG	human Chorionic Gonadotrophin
HIV	Human Immunodeficiency Virus
HPV	Human papillomavirus
HSV	Herpes simplex virus
IUI	Intra-Uterine Insemination
IVF	in vitro fertilization
ICSI	Intracytoplasmic Sperm Injection
IRPs	Iron Regulatory Proteins
STIs	Sexually Transmitted Infections
LPO	Lipid Peroxidation
MAGI	Male Accessory Gland Infection
mV	Millivolts
MESA	Microsurgical Epididymal Sperm Aspiration
MetS	Metabolic Syndrome
micro-TESE	Microsurgical Testicular Sperm Extraction
NOX5	NADPH Oxidase 5
Nrf2/HO-1	nuclear factor erythroid 2-related factor 2/heme oxygenase-1
OH^−^	Hydroxide Ion
ORP	Oxidation-Reduction Potential
OS	Oxidative Stress
PBS	Phosphate-Buffered Saline
PDE5i	Phosphodiesterase type 5 inhibitors
PESA	Percutaneous Epididymal Sperm Aspiration
RLU	Relative Light Units
ROS	Reactive Oxygen Species
ROS-TAC	Reactive Oxygen Species–Total Antioxidant Capacity Score
SARS-CoV-2	Severe Acute Respiratory Syndrome Coronavirus 2
sORP	Semen Oxidation-Reduction Potential
SOD	Superoxide Dismutase
SERMS	Selective Estrogen Receptor Modulators
SGLT2i	Sodium-Glucose Cotransporter 2 Inhibitors
TAC	Total Antioxidant Capacity
TESA	Testicular Sperm Aspiration
TESE	Testicular Sperm Extraction
UTIs	Urinary Tract Infections
VDR	Vitamin D Receptor
Vitamin B:	
B6	Pyridoxine
B9	Folate
B12	Cobalamin
Vitamin C	Ascorbic acid
Vitamin D	Cholecalciferol (D_3_), Ergocalciferol (D_2_)
Vitamin E:	
Natural Isoforms	α-tocopherol, β-tocopherol, γ-tocopherol, δ-tocopherol
Tocotrienols	α, β, γ, δ-tocotrienol
WBC	White Blood Cells
WHO	World Health Organization
